# Transrectal ultrasound for intraoperative interstitial needle guidance in cervical cancer brachytherapy

**DOI:** 10.1007/s00066-024-02207-9

**Published:** 2024-02-26

**Authors:** J. Knoth, A. Sturdza, A. Zaharie, V. Dick, G. Kronreif, N. Nesvacil, J. Widder, C. Kirisits, M. P Schmid

**Affiliations:** 1 Department of Radiation Oncology, Comprehensive Cancer Center, Medical University of Vienna, Währinger Gürtel 18–20, 1090 Vienna, Austria; 2 Austrian Center for Medical Innovation and Technology, Wiener Neustadt, Austria

**Keywords:** Female urogenital diseases, Cervical neoplasms, Image-guided radiotherapy, TRUS, Image-guided adaptive brachytherapy

## Abstract

**Objective:**

This study aimed to prospectively assess the visibility of interstitial needles on transrectal ultrasound (TRUS) in cervical cancer brachytherapy patients and evaluate its impact on implant and treatment plan quality.

**Material and methods:**

TRUS was utilized during and after applicator insertion, with each needle’s visibility documented through axial images at the high-risk clinical target volume’s largest diameter. Needle visibility on TRUS was scored from 0 (no visibility) to 3 (excellent discrimination, margins distinct). Quantitative assessment involved measuring the distance between tandem and each needle on TRUS and comparing it to respective magnetic resonance imaging (MRI) measurements. Expected treatment plan quality based on TRUS images was rated from 1 (meeting all planning objectives) to 4 (violation of High-risk clinical target volume (CTV_HR_) and/or organ at risk (OAR) hard constraints) and compared to the final MRI-based plan.

**Results:**

Analysis included 23 patients with local FIGO stage IB2-IVA, comprising 41 applications with a total of 230 needles. A high visibility rate of 99.1% (228/230 needles) was observed, with a mean visibility score of 2.5 ± 0.7 for visible needles. The maximum and mean difference between MRI and TRUS measurements were 8 mm and –0.1 ± 1.6 mm, respectively, with > 3 mm discrepancies in 3.5% of needles. Expected treatment plan quality after TRUS assessment exactly aligned with the final MRI plan in 28 out of 41 applications with only minor deviations in all other cases.

**Conclusion:**

Real-time TRUS-guided interstitial needle placement yielded high-quality implants, thanks to excellent needle visibility during insertion. This supports the potential of TRUS-guided brachytherapy as a promising modality for gynecological indications.

**Supplementary Information:**

The online version of this article (10.1007/s00066-024-02207-9) contains supplementary material, which is available to authorized users.

## Introduction

Currently, standard of care in the treatment of locally advanced cervical cancer is radiochemotherapy including brachytherapy [1]. During the past two decades, the performance of brachytherapy has been substantially improved by the integration of MRI into treatment planning and by the development of combined intracavitary/interstitial applicators. Use of MRI enables precise visualization of an adaptive target volume, whereas combined intracavitary/interstitial applicator systems allow the dose to be shaped accordingly. The main advantage of these applicators is that in addition to the standard intracavitary applicator (e.g., tandem/ring or tandem/ovoid), interstitial needles can be inserted through predefined holes in the ring or ovoid into more lateral parts of the cervix and/or parametria. However, the optimal workflow and in particular the most adequate utilization of the numerous needle positions for an individual patient remain unclear. The standard procedure is to choose the positions based on clinical estimation guided by gynecological examination and any prebrachytherapy MRI scans, if available. As the needles are directly linked to the intracavitary applicator, correct positioning of the tandem is of utmost importance. Nevertheless, even with optimal preparation such as full MRI-based preplanning [2] with use of 3D-printed applicators, uncertainty remains intraoperatively regarding whether the needles have actually reached the intended locations. Therefore, there has recently been increasing interest in the intraoperative use of transabdominal [3] and transrectal ultrasound (TRUS) in gynecological brachytherapy

TRUS has been proven to be noninferior to MRI in terms of tumor and brachytherapy target volume assessment for cervical cancer, albeit until now with major limitations in applicator depiction [4–7]. While real-time needle visualization and guidance is standard of care in prostate cancer, it is still in its infancy for gynecological brachytherapy. The aim of the current study is to analyze visibility and spatial differences of interstitial needles in TRUS in comparison to MRI in patients treated with magnetic resonance image-guided adaptive brachytherapy (MR-IGABT) for cervical cancer with combined intracavitary/interstitial applicators and to predict the expected treatment plan quality by TRUS.

## Materials and methods

### Patients and treatment

This is a prospective single-arm cohort study which was approved by the institutional ethics committee. Patients were eligible if they had (1) biopsy-proven cervical cancer with a local FIGO stage IB-IVA, (2) treatment with MR-IGABT, (3) utilization of a combined intracavitary/interstitial approach, and (4) written informed consent. The overall treatment comprised pelvic external beam radiotherapy (45 Gy in 25 fractions of 1.8 Gy) with concomitant chemotherapy (cisplatin 40 mg/m^2^ body surface for up to five weekly cycles) followed by MR-IGABT. Brachytherapy was delivered at the end of treatment typically over four fractions administered within two applications aiming at a total dose of > 90 Gy EQD2 (equieffective dose, reference dose 2 Gy per fraction, linear quadratic model, α/β = 10 Gy) to 90% (D_90_) of CTV_HR_. Venezia-type applicators (Elekta AB, Stockholm, Sweden) with commercially available interstitial obturator-bound plastic needles were used for target coverage and dose delivery. The obturators consisted of stainless steel and tungsten. Interstitial needles were inserted through predefined template positions within the applicator ring in a straight and/or oblique orientation as well as “free-hand” without attachment to the applicator. Straight insertion is defined as parallel to the tandem in the axis of the uterus. Oblique insertion is defined as diagonally from the ring directed to the parametrial tissue with an angle of 20° to the tandem. Free-hand needles were typically implanted either laterally to the ring into the middle/distal parametrium for regions not reachable with predefined needle positions or anteriorly to the ring in the periurethral area.

### Transrectal ultrasound

**Fig. 1:**
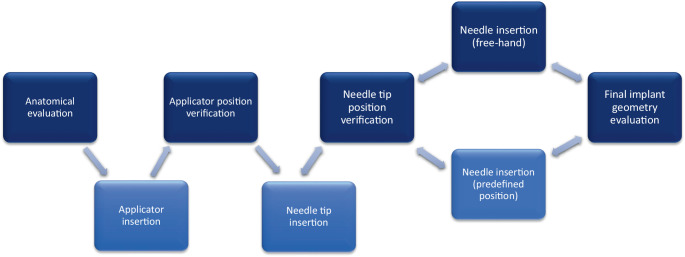
Process workflow describing intraoperative procedures. Steps involving transrectal ultrasound (TRUS) are colored in *dark blue*, steps without TRUS are colored in *light blue*

**Fig. 2:**
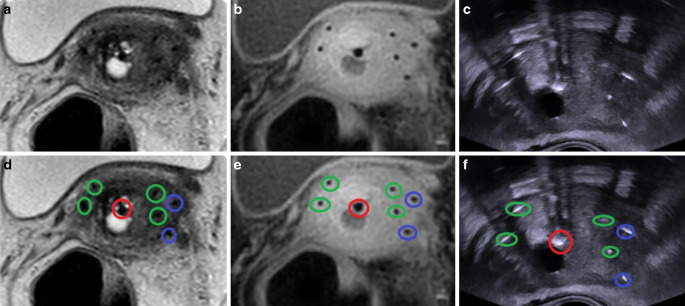
Example of a cervical cancer patient with the applicator as well as four straight and two oblique needles in place. The T2-weighted axial MRI in **a** and **d**, T1-weighted axial MRI in the **b** and **e**, and the corresponding axial transrectal ultrasound image in **c** and **f**. Tandem (*red*), straight needles (*green*), and oblique needles (*blue*) are marked in **d**–**f**

Free-hand ultrasound examination without use of a stepper unit was performed intraoperatively by a single investigator (= the treating physician) using a biplane transrectal probe (bk3000; BK Medical, Burlington, MA, USA) while the patients were in lithotomy position and under anesthesia following a dedicated in-house standard protocol. The high-resolution biplane transrectal probe (E14CL4b) has an image field sector of 138° in transverse view with a frequency range of 6–12 MHz in the herein applied scanning mode (B-mode) and a focal range of 3–60 mm. Our standard settings include a frequency range of 6 MHz and a focal range of 60 mm. The in-house standard protocol includes a first TRUS scan before applicator insertion for assessment of anatomy and expected target volume. Consecutive scans were then performed during or after tandem insertion and for each inserted needle separately (starting with the most anterior needles to avoid artefacts). The visualized target volume and the position of the first needle then determined the choice for the position of the subsequent ones. For predefined standard positions (straight or oblique) within the applicator, needle insertion and scanning were usually done sequentially (i.e., insertion of the needle for approximately one cm without TRUS – TRUS needle position check – if acceptable, further insertion until intended depth offline; if not acceptable, selection of a presumably more appropriate position) and only in critical situations (e.g., large vessels or bowel in direct proximity) or for free-hand needles simultaneously (i.e., insertion of needle below mucosa–TRUS position check and further insertion with online TRUS visualization). A final scan was done as soon as a satisfactory implant was reached to evaluate the overall implant geometry and to take representative screenshots. A workflow describing intraoperative procedures is also given in Fig. 1. CTV_HR_, applicator, and interstitial needles (always with the obturator in place) were visualized on both transverse and sagittal images. Only transverse images were used for image evaluation. An example of a TRUS image is given in Fig. 2.

### MRI

MRI was performed using an Ingenia 1.5T machine (Philips, Amsterdam, the Netherlands) according to the Gyn Groupe Européen de Curiethérapie and the European Society for Radiotherapy and Oncology (GEC-ESTRO) recommendations for imaging in IGABT [8]. T2-weighted axial and para-axial images were obtained covering at least the whole uterine fundus to the caudal border of the pubic bone including any vaginal tumor extension with the applicator in place for image analysis and treatment planning. Slice thickness was 3 mm. An example of MRI is given in Fig. 2.

### Image analysis

The primary aim of this analysis was the prospective assessment of quantitative and qualitative needle visibility on TRUS images in comparison to MRI. Therefore, the TRUS transducer was manually moved along the longitudinal axis of the uterus to locate the maximum axial diameter of the CTV_HR_ and to perform the intended measurements at this height. All image datasets were evaluated in transverse view in the axis of the uterus at the level of the largest CTV_HR_ diameter. At this level, transverse images were analyzed in regard of (1) needle visibility (yes vs. no); (2) distance of each needle to the tandem in millimeters; (3) distance of each needle to the outer border of the CTV_HR_ in millimeters; and (4) qualitative visibility, which was scored as follows: 0 = no visibility; 1 = poor discrimination, needle surface blurred; 2 = fair discrimination, needle surface indistinct; 3 = excellent discrimination, needle surface distinct. Each available needle position was systematically labelled for exact identification and subsequent comparison. The distance of each needle to the tandem and to the outer boarder of the CTV_HR_ was then measured on the respective MRI in the same manner as described above for comparison. Furthermore, the expected treatment plan quality based on implant geometry visualized by TRUS imaging was rated with the following scoring system: 1 = excellent (CTV_HR_ and OAR soft constraints expected to be met), 2 = sufficient (CTV_HR_ or OAR soft constraints expected to be violated), 3 = poor (CTV_HR_ and OAR soft constraints expected to be violated), 4 = insufficient (CTV_HR_ and/or OAR hard constraints expected to be violated), and compared to the respective final clinically approved MRI-based treatment plan. The final MRI-based treatment plan quality was rated with the following scoring system: 1 = excellent (CTV_HR_ and OAR soft constraints were met), 2 = sufficient (CTV_HR_ or OAR soft constraints were violated), 3 = poor (CTV_HR_ and OAR soft constraints were violated), 4 = insufficient (CTV_HR_ and/or OAR hard constraints were violated). The underlying soft and hard constraints were based on the EMBRACE II study protocol [9].

TRUS and MRI evaluation and measurements were performed by three radiation oncologists with more than 5 years of experience. To reduce a potential bias, all TRUS images were evaluated intraoperatively or directly after, without knowledge of subsequent MRI findings. The results of the measurements were promptly (during or immediately after the procedure) documented by the examiners using the case report form (CRF; see supplementary material).

### Statistical analysis

Descriptive statistics using SPSS statistics (version 24, IBM, Armonk, NY, USA) were done to calculate maximum (max), minimum (min), median, mean ± standard deviation (SD) for distance and visibility score for all needles as well as for straight, oblique, and free-hand needles separately. Paired t‑tests were performed to test for statistically significant differences between MRI and TRUS. Significance is defined as *p* < 0.05 after two-sided testing.

## Results

Between 05.2022 and 12.2022, 23 patients with 41 applications and a total of 230 needles (186 straight through the ring, 34 oblique through the ring, 10 free-hand insertions) were included in this study. The Tumor/Node/Metastasis (TNM) T stages consisted of T1b1 (*n* = 1), T2b (*n* = 18), T3b (*n* = 2), and T4 (*n* = 2, both with urinary bladder infiltration). The treated CTV_HR_ at the time of brachytherapy had a mean ± SD volume of 33.2 cm^3^ ± 12.9 cm^3^ (range 11–63 cm^3^, median 31 cm^3^). The median number of implanted needles per patient was *n* = 6 (range 3–12), a median of 5 needles (range 2–9) per patient were used for treatment. In total, 197/230 needles (86%) were used for treatment.

Overall, 228/230 needles (99.1%) were visible. Mean visibility score ± SD was 2.5 ± 0.7 for all visible needles. The respective results for straight, oblique, and free-hand needles did not differ and are shown in Table 1. The two non-visible needles (both free-hand inserted) were masked by artefacts or outside the field of view.

**Table 1:** Max, min, median, mean ± SD for visibility score, distance to tandem in millimeters on transrectal ultrasound (TRUS) at maximum target diameter, distance to tandem in millimeters on MRI at maximum target diameter, and difference between TRUS and MRI in millimeters at maximum target diameter; displayed for all visible needles as well as for visible straight, oblique, and “free-hand” needles separately

		Max	Min	Median	Mean	SD
All needles (*n* = 230)	Visibility	3	1	3	2.5	0.7
	TRUS	34	6	16.5	17.6	3.7
	MRI	35	4	17	17.8	4.0
	Difference	8	−4	0	−0.1	1.6
Straight needles (*n* = 186)	Visibility	3	1	3	2.5	0.6
	TRUS	23	13	16	16.4	1.8
	MRI	23	13	16	16.5	1.6
	Difference	4	−4	0	−0.1	1.3
Oblique needles (*n* = 34)	Visibility	3	1	3	2.6	0.7
	TRUS	30	21	24	24.2	2.2
	MRI	30	19	25	24.8	2.6
	Difference	8	−4	−1	−0.6	2.3
Free-hand needles (*n* = 8)	Visibility	3	1	3	2.5	0.8
	TRUS	34	6	18.5	18.3	9.9
	MRI	35	4	21	18.9	9.9
	Difference	6	−2	2	1.8	2.5

Max, min, mean ± SD distance of the visible needles to tandem was 35 mm, 4 mm, 17.8 mm ± 4.0 mm on MRI and 34 mm, 6 mm, 17.6 mm ± 3.7 mm on TRUS, respectively. Max and mean ± SD difference between MRI and TRUS were 8 mm and −0.1 mm ± 1.6 mm, respectively, which was not significant (*p* = 0.39). There was also no significant difference regarding straight (*p* = 0.41), oblique (*p* = 0.15), and free-hand needles (*p* = 0.09) separately. The respective results are summarized in Table 1. A difference of more than 3 mm between TRUS and MRI was found in 8 of 228 needles (3.5%).

The distance between tandem and the outer border of the CTV_HR_ at its widest point showed no significant difference between TRUS and MRI (mean ± SD of 1.9 mm ± 2.2 mm, *p* = 0.26). Straight needles had a mean distance of 5.3 mm ± 5.5 mm to the outer border of the CTV_HR_ on TRUS, whereas oblique needles showed a mean distance of 3.0 mm ± 2.7 mm.

The expected plan quality based on TRUS imaging and actual plan quality after MRI-based planning are compared in Table 2 and showed concordance in 28/41 implants (68%). Seven implants were underestimated by TRUS and six overestimated.

**Table 2:** Comparison of expected implant quality based on transrectal ultrasound (TRUS) images and actual implant quality after MRI-based planning

		Expected implant quality on TRUS			
		Excellent	Sufficient	Poor	Insufficient
Actual implant quality after MRI-based planning	Excellent	23	7	0	0
	Sufficient	6	3	0	0
	Poor	0	0	1	0
	Insufficient	0	0	0	1

## Discussion

This study prospectively investigated quantitative and qualitative needle visibility on TRUS in comparison to MRI in the treatment of locally advanced cervical cancer with combined intracavitary/interstitial MR-IGABT utilizing Venezia-type applicators as part of curative radiochemotherapy. Almost all needles (99%) were identifiable on TRUS, no matter which route of insertion (straight or oblique) was used. This contrasts with a previously published retrospective analysis [10] in which 87% of straight needles and only 51% of oblique needles could be detected on TRUS. The better detectability in the prospective study might be explained by the use of a different ultrasound device with slightly better image quality but, above all, by a different approach used for image acquisition. The TRUS probe was used free-handedly within the prospective study, thereby enabling the investigators to actively search and follow the needles in order to accurately adapt to the individual patient anatomy, while in the previous study, the TRUS probe was fixated by a stepper unit with a limited degree of freedom, causing more artefacts by, e.g., air bubbles. Furthermore, the distal parts of the parametrial space were partly outside the field of view with use of the stepper unit. Not only quantitative but also qualitative needle visibility improved from the retrospective (mean visibility score 1.4 ± 0.5 SD) to prospective analysis (mean visibility score 2.5 ± 0.7 SD). This might again be explained by the free-handed use of the TRUS probe, which allowed a closer proximity between probe and needle. Another important aspect could be found in the different needle material (titanium in the retrospective study vs. plastic [+ obturator] in the prospective study), which might impact on the extent of acoustic shadowing behind the needle, particularly in cases that require a higher number of needles for target coverage. The difference in distance between needles and tandem comparing TRUS and MRI is slightly better when comparing the current prospective assessment (mean ± SD: −0.1 ± 1.6 mm, 97% within a difference of 3 mm) to the retrospective study (mean ± SD: −0.3 ± 2.6 mm, 89% within a difference of 3 mm). Similarly, Rodgers et al. reported on visibility of all needles and a point difference of less than 3 mm in 78% of needles when using transvaginal ultrasound [11]. Comparing those differences to proposed uncertainties of less than 2 mm in quality assurance phantom tests [12], the clinical in vivo situation does not exceed this value by more than 1 mm in most cases.

Our findings show that the spatial relation of visible needles to the applicator is depicted equivalently on TRUS and MRI, which strongly suggests using TRUS for real-time needle guidance. Benefits of this approach are prompt guidance, evaluation, and confirmation of needle positions directly in the operating room (OR) leading to improved implant quality before the patient is transferred to the MRI facility. As a consequence, additional workload, time, and costs might be saved by avoiding repetitive transfers between OR and MRI.

The relevance of TRUS for IGABT is also reflected in the congruence of estimated implant quality based on TRUS imaging compared to actual implant quality after MRI-based treatment planning with regard to OAR and target dose constraints. In detail, three investigators with more than 5 years of experience in cervical cancer brachytherapy rated and, if necessary, adapted the implant in real time using TRUS imaging before MRI was performed. This led to excellent or sufficient implant quality in most cases (39/41; 95%), with only two patients having poor and insufficient treatment plans. The patient with expected and actual poor plan quality had a violated soft constraint of the CTV_HR_ (7.4 Gy at 90% of CTV_HR_) at the first implant due to a myoma blocking a needle from further insertion, which would have led to a projected summed dose of 87 Gy EQD2_10Gy_ at 90% of the CTV_HR_ in total. The second implant was rotated to a different angle, enabling avoidance of the myoma and thereby leading to a higher D_90_ of the CTV_HR_ (summed dose of 90.1 Gy EQD2_10Gy_). However, the soft constraint of the bowel remained violated (summed D_2cm3_ of 74.6 Gy EQD2_3Gy_) due to proximity of a bowel loop to the CTV_HR_. In the patient with expected and actual insufficient plan quality, the hard constraint of the urinary bladder (summed D_2cm3_ of 92 Gy EQD2_3Gy_) was violated due to urinary bladder infiltration. This was accepted in favor of sufficient target coverage. Both patients are currently without evidence of disease and without symptoms >G2 at 10 and 8 months post brachytherapy, respectively.

In prostate cancer brachytherapy, TRUS-based needle reconstruction and treatment planning is already possible [12–15]. In contrast, TRUS is suitable for target visualization and tandem and needle depiction in gynecological brachytherapy, but TRUS-only planning is not possible at the moment due to incomplete depiction of the surrounding OARs and uncertainties in applicator reconstruction. A possibility to overcome these limitations would be computed tomography (CT) (for OAR delineation) and TRUS (for target delineation) image fusion with automized applicator reconstruction by applicator tracking. Although this workflow was successfully tested with use of optical tracking, there were various disadvantages, with optical tracking limiting broad clinical implementation [7, 16]. Electromagnetic tracking (EMT) has been proven valuable to display, confirm, and follow needle positions in breast and prostate brachytherapy [17–22], and might also offer an attractive solution for cervical cancer brachytherapy. A prospective study to investigate EMT tracking for cervical cancer brachytherapy is currently under preparation in our department.

Strengths of this prospective investigation can be found in the number of patients and the high number of used needles (*n* = 230) in particular. To the best of our knowledge, this is the first study to investigate the visibility of different routes of needle insertion prospectively on TRUS. A new aspect is the estimation of implant quality (in the sense of “usability”), which might help to ease the whole treatment process. Lastly, the close local and temporal proximity between application of TRUS and MRI minimized the risk of needle or applicator movement between the two imaging modalities. Limitations might be found in the use of only a single applicator type and in the limited number and thereby limited data on free-hand needles.

## Conclusion

In this cohort, use of TRUS for real-time interstitial needle guidance resulted in high-quality implants due to excellent visibility of needles during insertion, thereby rendering TRUS-guided brachytherapy a promising modality to pursue and develop for gynecological indications.

### Supplementary Information

The Supplementary Information contains the case report form.

## Abbreviations

CRF: Case report form
CTV_HR_: High-risk clinical target volume
EQD2: Equieffective dose, reference dose 2 Gy per fraction
GEC-ESTRO: Groupe Européen de Curiethérapie and the European Society for Radiotherapy and Oncology
Max: Maximum
Min: Minimum
MRI: Magnetic resonance imaging
MR-IGABT: Magnetic resonance image-guided adaptive brachytherapy
OAR(s): Organ(s) at risk
OR: Operating room
SD: Standard deviation
TRUS: Transrectal ultrasound

## References

[1] Cibula D et al (2023) ESGO/ESTRO/ESP Guidelines for the management of patients with cervical cancer—Update 2023. Int J Gynecol Cancer 1;33(5):649–666 (May) 10.1136/ijgc-2023-004429 PMC10176411 37127326

[2] Fokdal L et al (2013) Clinical feasibility of combined intracavitary/interstitial brachytherapy in locally advanced cervical cancer employing MRI with a tandem/ring applicator in situ and virtual preplanning of the interstitial component. Radiother Oncol 107(1):63–68 (Apr) 23452917 10.1016/j.radonc.2013.01.010

[3] Van Dyk S et al (2009) Conformal brachytherapy planning for cervical cancer using transabdominal ultrasound. Int J Radiat Oncol Biol Phys 1;75(1):64–70 (Sep) 19250767 10.1016/j.ijrobp.2008.10.057

[4] Epstein E et al (2013) Early-stage cervical cancer: Tumor delineation by magnetic resonance imaging and ultrasound—A European multicenter trial. Gynecol Oncol 128(3):449–453 (Mar) 23022593 10.1016/j.ygyno.2012.09.025

[5] Schmid MP et al (2013) Feasibility of transrectal ultrasonography for assessment of cervical cancer. Strahlenther Onkol 189(2):123–128 (Feb) 23255091 10.1007/s00066-012-0258-1

[6] Schmid MP et al (2016) Transrectal ultrasound for image-guided adaptive brachytherapy in cervix cancer—An alternative to MRI for target definition? Radiother Oncol 120(3):467–472 (Sep) 26921168 10.1016/j.radonc.2016.01.021

[7] Smet S et al (2020) Hybrid TRUS/CT with optical tracking for target delineation in image-guided adaptive brachytherapy for cervical cancer. Strahlenther Onkol 196(11):983–992 (Nov) 32621011 10.1007/s00066-020-01656-2 PMC7653783

[8] Dimopoulos JC et al (2012) Recommendations from Gynaecological (GYN) GEC-ESTRO Working Group (IV): Basic principles and parameters for MR imaging within the frame of image based adaptive cervix cancer brachytherapy. Radiother Oncol 103(1):113–122 (Apr) 22296748 10.1016/j.radonc.2011.12.024 PMC3336085

[9] Pötter R., et al.; The EMBRACE II study: The outcome and prospect of two decades of evolution within the GEC-ESTRO GYN working group and the EMBRACE studies. Clin Transl Radiat Oncol. 2018 Jan 11;9:48–60. 10.1016/j.ctro.2018.01.001 PMC5862686 29594251

[10] Knoth J., et al.; Toward 3D-TRUS image-guided interstitial brachytherapy for cervical cancer. Brachytherapy. 2022 Mar-Apr;21(2):186–192. 10.1016/j.brachy.2021.10.005 34876361

[11] Rodgers JR et al (2019) Intraoperative 360-deg three-dimensional transvaginal ultrasound during needle insertions for high-dose-rate transperineal interstitial gynecologic brachytherapy of vaginal tumors. J Med Imaging (bellingham) 6(2):25001 (Apr) 30989088 10.1117/1.JMI.6.2.025001 PMC6451200

[12] Siebert FA et al (2020) GEC-ESTRO/ACROP recommendations for quality assurance of ultrasound imaging in brachytherapy. Radiother Oncol 148:51–56 (Jul) 32335363 10.1016/j.radonc.2020.02.024

[13] Batchelar D., et al.; Validation study of ultrasound-based high-dose-rate prostate brachytherapy planning compared with CT-based planning. Brachytherapy. 2014 Jan-Feb;13(1):75–9. 10.1016/j.brachy.2013.08.004 24080299

[14] Zheng D., et al.; A novel method for accurate needle-tip identification in trans-rectal ultrasound-based high-dose-rate prostate brachytherapy. Brachytherapy. 2011 Nov-Dec;10(6):466–73. 10.1016/j.brachy.2011.02.214 21549646

[15] Siebert FA et al (2009) Imaging of implant needles for real-time HDR-brachytherapy prostate treatment using biplane ultrasound transducers. Med Phys 36(8):3406–3412 (Aug) 19746773 10.1118/1.3157107

[16] Nesvacil N., et al.; Combining transrectal ultrasound and CT for image-guided adaptive brachytherapy of cervical cancer: Proof of concept. Brachytherapy. 2016 Nov–Dec;15(6):839–844. 10.1016/j.brachy.2016.08.009 27693172

[17] Poulin E et al (2015) Fast, automatic, and accurate catheter reconstruction in HDR brachytherapy using an electromagnetic 3D tracking system. Med Phys 42(3):1227–1232 (Mar) 25735278 10.1118/1.4908011

[18] Yang Z et al (2020) Verification of needle guidance accuracy in pelvic phantom using registered ultrasound and MRI images for intracavitary/interstitial gynecologic brachytherapy. J Contemp Brachytherapy 12(2):147–159 (Apr) 32395139 10.5114/jcb.2020.94583 PMC7207233

[19] Dehghan E et al (2018) EM-enhanced US-based seed detection for prostate brachytherapy. Med Phys 45(6):2357–2368 (Jun) 29604086 10.1002/mp.12894

[20] Bharat S et al (2014) Electromagnetic tracking for catheter reconstruction in ultrasound-guided high-dose-rate brachytherapy of the prostate. Brachytherapy Nov-dec 13(6):640–650 10.1016/j.brachy.2014.05.012 24929641

[21] Boutaleb S et al (2015) Performance and suitability assessment of a real-time 3D electromagnetic needle tracking system for interstitial brachytherapy. J Contemp Brachytherapy 7(4):280–289 (Aug) 26622231 10.5114/jcb.2015.54062 PMC4643737

[22] Beaulieu L et al (2018) Real-time electromagnetic tracking based treatment platform for high-dose-rate prostate brachytherapy: Clinical workflows and end-to-end validation. Brachytherapy. Jan 17(1):103–110 10.1016/j.brachy.2017.04.247 28576644

