# Supplementary material for: Transrectal ultrasound for intraoperative interstitial needle guidance in cervical cancer brachytherapy
Source: Strahlenther Onkol. 2024 Feb 26;200(8):684–90. doi: 10.1007/s00066-024-02207-9 (PMC11272749; doi:10.1007/s00066-024-02207-9)
Supplement: Supplementary file 1 — The Supplementary Information contains the case report form. [file 66_2024_2207_MOESM1_ESM.docx]

| **Needle position**  **(based on Venezia applicator)** | **Visibility on TRUS**  **0=no**  **1=yes** | **Qualitative visibility on TRUS (0-3*)** | **Distance from tandem at largest CTV_HR_ diameter on TRUS (in mm)** | **Distance from tandem at largest CTV_HR_ diameter on MRI (in mm)** | **Max. distance to border of CTV_HR_ on TRUS (in mm)** |
| --- | --- | --- | --- | --- | --- |
| 1A |  |  |  |  |  |
| 1B |  |  |  |  |  |
| 1C |  |  |  |  |  |
| 1D |  |  |  |  |  |
| 1E |  |  |  |  |  |
| 1F |  |  |  |  |  |
| 1G |  |  |  |  |  |
| 1H |  |  |  |  |  |
| 2A |  |  |  |  |  |
| 2B |  |  |  |  |  |
| 2C |  |  |  |  |  |
| 2D |  |  |  |  |  |
| 2E |  |  |  |  |  |
| 2F |  |  |  |  |  |
| 2G |  |  |  |  |  |
| 2H |  |  |  |  |  |
| Free-hand 1 |  |  |  |  |  |
| Free-hand 2 |  |  |  |  |  |
| Free-hand 3 |  |  |  |  |  |
| Free-hand 4 |  |  |  |  |  |

| **Patient ID** |
| --- |

**CRF TRUS**

*0=no visibility 1= poor, 2= fair, 3= excellent

| **Expected plan quality** | |
| --- | --- |
| (1 )Excellent (CTV_HR_ and OARs soft constraints met) |  |
| (2) Sufficient (CTV_HR_ or OARs soft constraints violated) |  |
| (3) Poor (CTV_HR_ and OARs soft constraints violated) |  |
| (4) Insufficient (CTV_HR_ and/or OARs hard constraints violated) |  |

**Comment (mandatory for case 2-4):**

**Max. peripheral distance from tandem (TRUS):**

**Max. peripheral distance from tandem (MRI):**
